# The Treatment of Cholecystitis and Cholelithiasis by Tibetan Medicine

**DOI:** 10.1155/2021/9502609

**Published:** 2021-09-30

**Authors:** Lin Pan, Jie Gao, Yunfeng Han, Yi Shi, Xi Tang, Lili Pu, Xianrong Lai, Renqing Dongzhu, Jinkui Zhang, Qieni Xiangmao, Jiumei Pengcuo

**Affiliations:** ^1^School of Pharmacy, Chengdu University of Traditional Chinese Medicine, Chengdu 611137, China; ^2^School of Ethnic Medicine, Chengdu University of Traditional Chinese Medicine, Chengdu 611137, China; ^3^Qinghai (Tso-Ngo) University Tibetan Medical College, Xining 810000, China; ^4^Qinghai Jiumei Tibetan Medicine Co., Ltd., Xining 810000, China

## Abstract

Cholecystitis and cholelithiasis is one of the factors threatening human health. It is very important to find drugs for the treatment of cholecystitis and cholelithiasis. Tibetan medicine is one of the traditional medical systems in China. It has rich experience in treating various diseases. This paper summarizes the treatment of cholecystitis and cholelithiasis through literature review of Tibetan medicine monographs, drug standards, Tibetan medicine, and prescriptions. In the Tibetan medicine system, 170 kinds of Tibetan medicine and 38 kinds of Tibetan prescriptions were found to treat cholecystitis and cholelithiasis. Among them, there are 35 modern researches related to the treatment of cholecystitis and cholelithiasis. Their names, families, medicinal parts, chemical constituents, and pharmacological activities are introduced in detail. These Tibetan medicines and prescriptions may be a precious gift of ancient Tibetan medicine to the world, and may also become potential drug candidates for the treatment of cholecystitis and cholelithiasis. Modern phytochemistry, pharmacology, metabonomics, and/or clinical trials can be used to confirm its medicinal value in the treatment of cholecystitis and cholelithiasis, identify active compounds, clarify its potential mechanism of action, and clarify its toxicity and side effects. This article provides a new idea and source for the treatment of cholecystitis and cholelithiasis.

## 1. Introduction

Cholecystitis and cholelithiasis are common diseases of the biliary system, which are induced by many factors, including emotion, inflammation, and diet. Cholelithiasis is the process of gallstone formation, and cholecystitis is an acute or chronic gallbladder infection [1], which may be related to gallstones. Studies have found that 95% of patients with acute cholecystitis also suffer from cholelithiasis [2, 3]. Cholecystitis and cholelithiasis usually have paroxysmal biliary colic, mostly in the right upper abdomen, and can radiate to the right shoulder, lasting for several hours as the main clinical manifestation [4], and may also be accompanied by nausea and vomiting. Epidemiological survey shows that the incidence rate of this disease in adults is about 10% to 15%, and that of women is significantly more than that of men. The age of onset is 40∼60 years. In addition, the incidence rate of gallstones is also related to geographical, ethnic, and dietary habits. With the improvement of living standards and changes in diet, the incidence rate of cholecystitis and cholelithiasis is increasing year by year, especially in economically developed areas [5–8]. At the same time, cholecystitis and cholelithiasis are also closely related to the occurrence of gallbladder cancer, pancreatic cancer, and colorectal cancer. Therefore, it is necessary to find an effective method for the treatment of cholecystitis and cholelithiasis. At present, the treatment of cholecystitis and cholelithiasis is mainly cholecystectomy and endoscopic or drug treatment of complications, but the postoperative recovery is not optimistic, and complications seriously affect the quality of life of patients [4]. Tibetan medicine has accumulated experience in the treatment of cholecystitis and cholelithiasis with little side effect, low cost, and improvement of overall symptoms.

**Figure 1:**
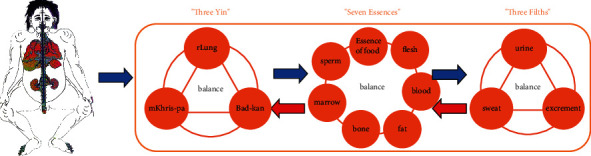
Balance of three yins, seven essences, and three filths in Tibetan medicine.

Cholecystitis and cholelithiasis belong to the category of “mKhris-pa”, “pimple”, “pimple tumor”, and “gall disease” in Tibetan medicine, which is the essence that does not disappear but falls from the liver to the gallbladder. According to the Tibetan classic *Rgyud bzhi*, the occurrence and development of diseases are related to three yins: rLung, mKhris-pa, and Bad-kan. Food essence, blood, meat, fat, bone, marrow, and semen are the seven essences of the human body. Sweat, urine, and feces produced by human excretion belong to filth, which are called three filths (Figure 1). Three yins dominate the movement of seven essences and three filths. Three yins, seven essences, and filths should maintain relative balance, depend on each other, and perform their own duties in order to maintain the normal physiological activities of the body. If the three change their position, shape, and capacity due to various internal and external factors, such as increase, loss, and disorder, they will lose their original balance and then cause physiological changes of the body, leading to the emergence of diseases [9]. Cholecystitis and cholelithiasis are caused by the essence and extrinsic causes of the essence of the human body. The dynamic balance of human body's “three yins”, such as rLung, mKhris-pa, and Bad-kan, is damaged, resulting in diseases that cannot digest and absorb the essence and flow to the biliary system and mix with bile and form the disease under the action of “rLung” [10–12].

Tibetan medicine believes that the human body is an interconnected whole, recorded in the ancient Tibetan medicine literature “*Blue Glaze*”, “There are four or five veins in the human body; according to the principle, it plays all the functions of the body and connects with each other, thus restricting all parts of the body, diseases, essence, filth. and so on. No matter what happens, it will not happen against the time”. The above-mentioned four kinds of dependent veins, with their respective positions as the axis, shoot out branches and sub-branches to the left, right, and middle, which are distributed in all parts of the human body and connected with all the tissues and organs of the human body, so that the three genes, seven matrixes, and three wastes of the human body can operate normally and maintain life activities. Therefore, in the treatment of cholecystitis and cholelithiasis, Tibetan medicine pays attention to the influence of drugs on the whole and balances the three genes. Tibetan doctors believe that the main cause of cholecystitis and cholelithiasis is the imbalance of three genes caused by diet and environmental problems. Therefore, Tibetan doctors put forward the treatment methods of cholecystitis and cholelithiasis from the aspects of medicine, external treatment, diet, daily life behavior, and so on.

According to statistics [13], 3105 kinds of natural medicines have been used in Tibetan medicine system, including 2644 kinds of plants, 321 kinds of animals, and 140 kinds of minerals. Tibetan medicine has accumulated experience in treating various diseases, especially chronic diseases such as cholecystitis, cholelithiasis, hepatitis, high altitude polycythemia, gastritis, stroke, and rheumatism. In fact, Tibetan medicine has been widely used in the treatment of cholecystitis and cholelithiasis, but these records are relatively loose and lack systematic summary, which is not conducive to the further application of Tibetan medicine in cholecystitis and cholelithiasis. This article summarizes the treatment of cholecystitis and cholelithiasis with Tibetan medicine from two aspects, the first is the Tibetan medicine from *Dictionary of Chinese Ethnic Medicine*, *Drug Standards of Tibetan Medicine*, *Blue Glaze*, *Jing Zhu Materia Medica*, *Chinese Materia Medica Tibetan Medicine Roll*, *Chinese Herbalism for Tibetan Medicine*, *Tibetan Medicine Annals*; the second is the prescriptions for treating cholecystitis and cholelithiasis in notes on common prescriptions of *Annotation of Commonly Used Tibetan Medicine Prescription*, *Drug Standards of Tibetan Medicine*, *Treasure House of Tibetan Medicine Prescriptions*, *Chinese Materia Medica Tibetan Medicine Roll*, *Chinese Herbalism for Tibetan Medicine*.

## 2. Materials and Methods

We searched *Jing Zhu Materia Medica*, *Dictionary of Chinese Ethnic Medicine*, *Drug Standards of Tibetan Medicine*, *Treasure House of Tibetan Medicine Prescriptions*, *Tibetan Medicine Annals*, *Chinese Herbalism for Tibetan Medicine*, and other Tibetan medicine monographs and drug standards, obtained Tibetan medicine and prescription information for the treatment of cholecystitis and cholelithiasis, name of species, families, Tibetan names, and medicinal parts have been recorded. The botanical names of original plants are mainly from the references, and verified through the “Flora of China (http://frps.eflora.cn/)” database based on their Chinese names. The database of “The Plant List (http://www.theplantlist.org/)” is also used to standardize their Latin names. In order to understand the most commonly used Tibetan medicine for the treatment of cholecystitis and cholelithiasis and obtain the use frequency of each drug, the Traditional Chinese Medicine Inheritance Support System (version 2.5) was used to manually input all the prescriptions collected into TCMISS software, and click the “frequency statistics” module to sort the use frequency of each drug from large to small. In addition, we searched Chinese online databases (e.g., Wanfang, VIP, and CNKI) and international databases (e.g., ISI's Web Science, GeenMedical, sci-hub) for selected species of active ingredients and biological or pharmacological effects, using their dialect, English, or Latin names to search for keywords [14].

## 3. Results

### 3.1. Literature Research Results of Tibetan Medicine in the Treatment of Cholecystitis and Cholelithiasis

**Figure 2:**
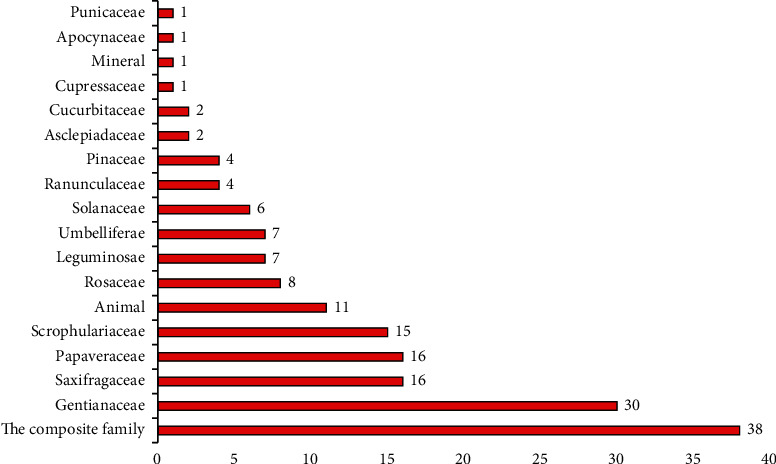
The most common families and genera of Tibetan medicine in the treatment of cholecystitis and cholelithiasis.

**Figure 3:**
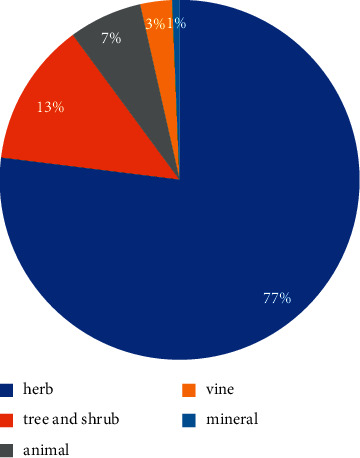
The most common source of Tibetan medicine for cholecystitis and cholelithiasis.

**Figure 4:**
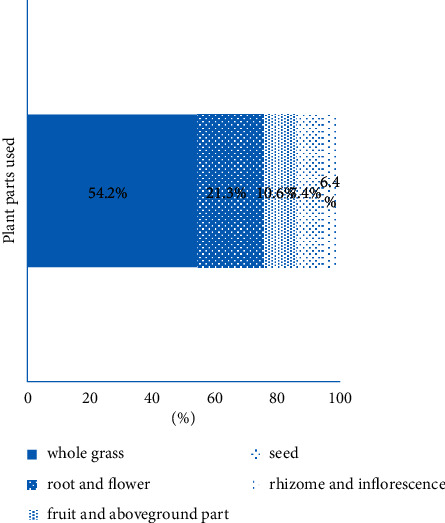
The most common plant parts used of Tibetan medicine for cholecystitis and cholelithiasis.

This paper records the application of 170 kinds of Tibetan medicine and 38 kinds of Tibetan prescriptions in the treatment of cholecystitis and cholelithiasis. These 170 Tibetan medicines are distributed in 24 families and 49 genera. The most common families were Compositae (22.4%), Gentianaceae (17.6%), Saxifragaceae (9.4%), Papaveraceae (9.4%), and Scrophulariaceae (8.8%) (Figure 2). In addition, herbs (77.0%) were the main sources of these Tibetan medicines, followed by trees or shrubs (12.9%), animals (6.5%), vines (2.9%), and finally minerals (0.6%) (Figure 3). Among the plant parts used in the treatment of cholecystitis and cholelithiasis, whole grass was the most used (54.2%), followed by roots and flowers (21.3%), fruits and aboveground parts (10.6%), followed by seeds (7.4%), and finally rhizomes and inflorescences (6.4%) (Figure 4).

Among 170 kinds of Tibetan medicine for cholecystitis and cholelithiasis, 35 are used in modern research related to cholecystitis or cholelithiasis. The scientific name, Chinese name, Tibetan name, family, medicinal parts, related modern research, and reported active parts of these 35 drugs are shown in Table 1. Among 170 kinds of Tibetan medicine for cholecystitis and cholelithiasis, 60 kinds have not been studied by modern research methods, and 75 kinds have not been used for modern treatment of cholecystitis or cholelithiasis. Therefore, for the further use of Tibetan medicine, i.e., for a better and safer use of these Tibetan medicines in the treatment of cholecystitis and cholelithiasis, it is necessary to conduct a more systematic, more in-depth, and more complete study.

It can be seen from the above table that the current treatment of cholecystitis and cholelithiasis by traditional Tibetan medicine mainly focuses on improving symptoms and reducing inflammation and pain, and the pharmacological effect focuses on anti-inflammatory and analgesic research. At the same time, it also has a certain inhibitory effect on the pathogenic bacteria related to cholecystitis and cholelithiasis. Some modern studies on drugs include the effects of bile secretion. Among the above 35 drugs, only bear bile and anisodamine can promote bile secretion, have stone dissolving effect, and improve bile composition. In the meantime, it can be seen from the above table that the active part of many Tibetan medicines is not a single component, but an extract, which may contain a variety of compounds, such as dandelion extract, triterpenoids, saponins, etc. At the same time, the mechanism of action of most Tibetan medicine has not been clarified, and the basic research and clinical research are still to be improved. Therefore, a more systematic and perfect research on Tibetan medicine can make the application of Tibetan medicine more safe and reliable. According to the analysis of Table 1, three kinds of Tibetan medicine for cholecystitis and cholelithiasis are selected for detailed analysis. The modern research of these three kinds of Tibetan medicine proves that the medicine can be applied to cholecystitis and cholelithiasis.

#### 3.1.1. Swertia Punicea

*Swertia punicea* Hemsl. (Latin name of original plant), also known as ཟངས་ཏིག (Tibetan name) and Zihong Zhangyacai (Chinese name) is the whole plant of Swertia of gentiaceae. It is recorded in *Chinese Herbalism for Tibetan Medicine* that it can clear liver and gallbladder heat, diuresis, treat “mKhris-pa” disease, blood disease, hepatitis, cholecystitis, various kinds of febrile diseases, and edema.

The main chemical constituents of Swertia Punicea include swertiamarin, mangiferin, oleanolic acid, other koyamanone, and iridoid [102]. It has liver protection, anti-inflammatory, antivirus, central nervous system protection, digestive tract protection, and hypoglycemia effects [17]. Peng et al. [103, 104] found that Swertia Punicea can significantly increase bile flow, increase bilirubin content in bile, and reduce serum bilirubin content in normal rats. They believed that Swertia Punicea has obvious cholagogic and promotes bilirubin excretion. Bhattacharya et al. [105] used the hot plate method, tail flick method, and acetic acid twist method to evaluate the analgesic effect of swertiflorin. The results showed that swertine had significant analgesic effect, a dual analgesic effect, i.e., peripheral and central. Saravanan et al. [106] used IL-1*β*. The anti-inflammatory effect of swertiflorin was investigated by fibroblast-like synovium cells. The results showed that swertine significantly inhibited IL-1*β*. The proliferation of synovial cells, the reduction of NO production, the decrease of caspase-3, inhibition of TNF*α*, IL-6, PGE2, COX-2, iNOS, MMP levels, and inhibition of p38 MAPK*α* occurred at the same time level. Saravanan et al. [107] established adjuvant arthritis model in rats and treated them with swerticin. The results showed that swertine caused dose-related inhibition of paw swelling and body mass of rats, decreased IL-1, IL-6, and TNF levels, and significantly increased the level of anti-inflammatory factors (IL-10 and IL-4). It was found that swertine can inhibit NF-KBP65 and p-I*κ*B*α* at both whole animal level and cellular level, the expression of p-JAK2 and p-STAT3 proteins confirmed that swerticrine may pass NF-*κ*B/I*κ*B, and the JAK2/STAT3 signaling pathway has anti-inflammatory effect.

In conclusion, Swertia Punicea has the pharmacological effects such as choledoch, anti-inflammatory, and analgesic effects, but the mechanism of its treatment of cholecystitis and cholelithiasis is unclear, and further modern research is needed.

#### 3.1.2. Saltpeter

*Nitrokalite* (Latin name of original plant), also known as ཟེ་ཚྭ། (Tibetan name), Xiao Shi (Chinese name), also called nitre is a nitrate type nitrite mineral, mainly containing potassium nitrate. *Jing Zhu Materia Medica*, the classic of Tibetan medicine, records that “Huo nitrate can eliminate stones, break stones, and break pimple tumor”, while *Medical Canon in Four Sections* records that its effect is to transform stones and cure pimple lump. It mainly contain potassium nitrate, and can treat cholelithiasis, urinary calculi, cholestatic hepatitis, angina pectoris of coronary heart disease, cysticercosis, and so on [108–110].

Yuan et al. [111], through clinical research, reported that 38 cases of urinary calculi were treated with huonite, and good curative effect was obtained. It was determined that huonite can treat calculi. Modern pharmacological research also considered that huonite has good anti-inflammatory and diuretic effects, can increase intestinal peristalsis, and exhibits cholagogic effects. The clinical observation of 90 cases of cholelithiasis treated with Saltpeter tablet shows that Saltpeter tablet has a certain dissolution effect on gallstones and intrahepatic bile duct stones. Pharmacological studies have proved that Saltpeter tablet can reduce the content of cholesterol in dog bile, and the total bile acid is relatively increased. It is believed that the Saltpeter tablet can accelerate the decomposition and metabolism of cholesterol, reduce the content of cholesterol in bile, increase the level of bile salt and phospholipid, and gradually dissolve the stones. At the same time, the study found that nitre tablet has a strong inhibitory effect on common pathogenic bacteria of the biliary tract and can promote the metabolism of gallbladder epithelium, and enhance its epithelial neogenesis, secretion, and excretion function [112, 113].

#### 3.1.3. Anisodus Tanguticus

*Anisodus tanguticus (Maxim.) Pascher* (Latin name of original plant), also known as ཐང་ཕྲོམ་ནག་པོ། (Tibetan name) and Shan Langdang (Chinese name) is anisodamine of Solanaceae. *Drug Standards of Tibetan Medicine* records that it treats acute abdominal pain, anthrax, gastroenteritis, biliary Ascaris, and cholelithiasis. It mainly contains anisodamine, anisodamine, anisodine, scopolamine, ormosine, tropine, atropine, and dehydrated atropine and other alkaloids [114, 115].

Anisodamine can significantly promote the spontaneous discharge of choledocholithiasis, and the effect of anisodamine is more significant for choledocholithiasis with diameter <5 mm [79]. Anisodamine can promote bile excretion and reduce serum endotoxin level, and its mechanism may be related to improving the function of sphincter of Oddi [81]. Anisodamine hydrobromide injection can promote the excretion of symptomatic CBD stones with single diameter ≤10 mm [82]. Anisodamine combined with ketorolac tromethamine in the treatment of patients with biliary colic caused by gallstones can improve the total effective rate and shorten the time of biliary colic and hospitalization time [116]. Clinical studies have found that atropine can relieve the pain of elderly patients with acute cholecystitis, with less adverse reactions after treatment and has a good effect on the patients' depression and anxiety [117]. In addition, clinical studies have found that atropine combined with dexamethasone has better effect on relieving biliary colic with rapid pain relief, high curative effect, low short-term recurrence rate, and less adverse reactions [118–121].

### 3.2. Literature Research on the Treatment of Cholecystitis and Cholelithiasis with Tibetan Prescription

**Figure 5:**
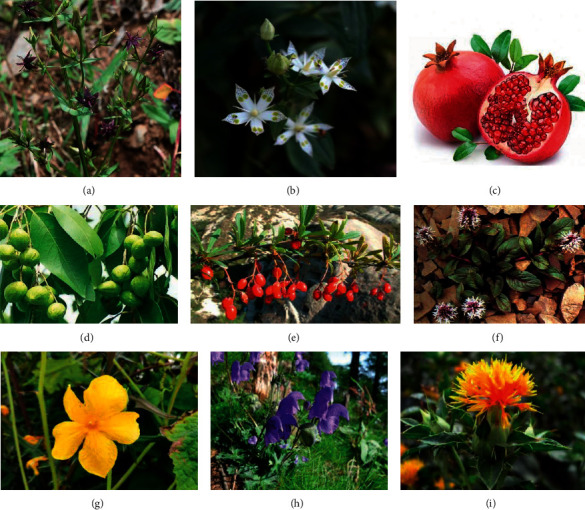
The picture of common Tibetan medicine in Tibetan prescriptions. (a) *Swertia mussotii* Franch. (b) *Swertia bimaculata* (Sieb. et Zucc.) Hook. f. et Thoms. ex C. B. Clark. (c) *Punica granatum* L. (d) *Terminalia chebula* Retz. (e) *Berberis poiretii* Schneid. (f) *Lagotis glauca* Gaertn. (g) *Herpetospermum pedunculosum* (Ser.) C. B. Clarke. (h) *Aconitum tanguticum* (Maxim.) Stapf. (i) *Carthamus tinctorius* L.

In order to understand the most commonly used Tibetan medicine in the treatment of cholecystitis and cholelithiasis, TCMISS software was used for data mining to obtain the use frequency of Tibetan medicine in traditional Tibetan prescriptions.  Figure 5 shows the picture of common Tibetan medicine in Tibetan prescriptions. Thirty-eight prescriptions for cholecystitis and cholelithiasis were collected from Tibetan medicine monographs and drug standards. In the treatment of cholecystitis and cholelithiasis, the top ten Tibetan medicines were Swertia mussotii (25 times), Herpetospermum pedunculosum (24 times), Terminalia chebula (19 times), pomegranate (14 times), Aconitum tanguticum (14 times), Yanjing (13 times), Lagotisglauca (13 times), Berberidis Cortex (11 times), Carthami Flos (11 times), and Heibingpian (10 times). Among the ten Tibetan medicines mentioned above, Swertia mussotii, pomegranate, Aconitum tanguticum, Berberidis Cortex, and Carthami Flos have been proved by modern research to have the potential to treat cholecystitis and cholelithiasis and can be used as potential drugs. Among the 38 kinds of Tibetan medicine prescriptions for cholecystitis and cholelithiasis, two of them have been proved to be able to be used in the treatment of cholecystitis and cholelithiasis in modern research. The following is a detailed introduction of these two kinds of Tibetan medicine.

#### 3.2.1. Shiwei Heibingpian Pill

Shiwei Heibingpian pill is a Tibetan medicine named གར་ནག་བཅུ་པ། (kanajiubaribu). The prescription comes from *Medical Canon in Four Sections*, included in the Ministry of Health drug standard Tibetan medicine (No.: WS3-BC-0215-95). According to the *Complete Collection of Tibetan Medicine*, the specific formula of this prescription is as follows: Heibiingpian 150 g, pomegranate seed 150 g, cinnamon 35 g, cardamom 20 g, Piper longum 25 g, Terminalia chebula 100 g, Guangming salt 20 g, Herpetospermum caudigerum 25 g, semen holarrhenae 20 g, bear gall 1.5 g. Shiwei Heibingpian pill can be used for warming stomach and eliminating food, breaking accumulation, and promoting gall bladder. It is used for treating lung diseases, indigestion, nausea, Bad-kan's tumor, cholecystitis, gallstone, cold gall disease, and jaundice.

Tibetan medicine Shiwei Heibingpian pill is commonly used in the treatment of gallstones in clinic. It can effectively inhibit inflammation and bacteria, promote bile secretion, reduce the occurrence of polyps and gallstones, and has good analgesic effect [122]. Yuan [123] found that the holistic nursing mode was adopted in the treatment of chronic cholecystitis cholelithiasis in the course of laparoscopic cholecystectomy combined with Shiwei Heibingpian pill, which is more conducive to improve the nursing effect and patient satisfaction, reduce the probability of complications, promote recovery, shorten the hospitalization time, and reduce the economic burden. Yang et al. [124–127] found that Shiwei Heibingpian pill, Shiyiwei Hezi pill, and Poliu Yuejing powder can improve the antibacterial ability, increase bile secretion, promote gallbladder contraction, relax biliary sphincter, and other effects, so as to reduce or even eliminate inflammation and discharge stones. Siqingtu Nala et al. [128] found that Shiwei Heibingpian has obvious inhibitory effect on Helicobacter pylori.

To sum up, Shiwei Heibingpian pill has been proved to be able to treat cholecystitis and cholelithiasis in clinical studies, but its mechanism of action remains to be found, and further study is needed to clarify its mechanism of action, so as to facilitate its better application in disease treatment.

#### 3.2.2. Bawei Zhangyacai Powder

Bawei Zhangyaccai powder is a Tibetan medicine named ཏིག་ཏ་བརྒྱད་པ། (didajieba). The prescription comes from Tibetan doctor doudajieba and is included in the Ministry of Health drug standard Tibetan medicine (No.: WS3-BC-0242-9). The specific formula of the prescription is as follows: Swertia mussotii 300 g, Lagotis glauca 200 g, Herpetospermum caudigerum 80 g, Hypecoum erectum 200 g, Tanggute Aconitum 200 g, Berberidis Cortex 160 G, Cicerbita azurea 240 g, and banksia rose 200 g. Bawei Zhangyaccai powder is used for cholecystitis and jaundice hepatitis.

Wang [129] used Bawei Zhangyaccai powder for clinical treatment of 80 patients with chronic cholecystitis, and found that except for 1 case who had something to go out and could not evaluate the curative effect, all of them were effective, including 72 cases with obvious pain relief after 3 days of treatment, 64 cases were cured after 1 course of treatment, 14 cases were cured after 2 courses of treatment, and the recurrence rate was reduced at the same time. Na et al. [130] found that Bawei Zhangyaccai powder has anti-CCl_4_ liver injury effect, and its mechanism may be related to increasing IL-10 and inhibiting TNF-*α*.

## 4. Deficiencies and Prospects

Cholecystitis and cholelithiasis belong to the category of “mKhris-pa”, “pimple”, “pimple tumor”, and “gall disease” in Tibetan medicine. This paper summarizes the drugs in classic works of Tibetan medicine for cholecystitis and cholelithiasis. There are still quite a few drugs that can treat “cholelithiasis”, “cholepathy”, “ruffian tumor”, “mKhris-pa”, and “gall fever”. Besides the Tibetan medicine, which can directly explain the treatment of cholecystitis and cholelithiasis, 356 Tibetan drugs can be used to treat the above diseases. These drugs can be used as treatment of cholecystitis. The potential drugs of cholelithiasis also have further research value. For example, Berberidis Cortex is the dry endothelium of many Berberis plants in Berberidaceae (such as *Berberis dictyophylla* Franch.). The classic Tibetan medicine *Blue Glaze* records “clearing kidney heat”, and *Jing Zhu Materia Medica* records “Berberidis Cortex collects various poisons and dries yellow water”. It mainly contains alkaloids, such as berberine, Magnolia alkaloid, jatrorrhizine, palmatine, etc. [131], which can be used to treat dysentery, urinary tract infection, yellow water disease, eye disease, nephritis, and other diseases [132, 133]. Some studies have found that berberine, the main component of Berberidis Cortex, can reduce serum cholesterol by up regulating the expression of LDLR in hepatocytes. It can reduce the expression of COX-2 and the formation of cholesterol crystals. It can reduce the inflammation of gallbladder, reduce the formation of cholesterol crystals, and reduce the occurrence of cholesterol stones. It can reduce the cholesterol content in plasma and bile, increase the bile acid content, reverse the trend of gallstone formation, and achieve the purpose of preventing gallstone. At the same time, berberine hydrochloride can inhibit intestinal cholesterol uptake, inhibit secondary bile acid synthesis, regulate bile acid metabolism, and inhibit gallstone formation through remodeling intestinal flora [134–136]. It shows that these drugs may also have the effect of treating cholecystitis and cholelithiasis, which should be further studied in order to better benefit the treatment of the disease.

At the same time, we should pay attention to the therapeutic effect of Tibetan medicine on cholecystitis and cholelithiasis, and also pay attention to the potential toxicity of Tibetan medicine. For example, the literature records that Aconitum tanguticum has small toxicity, and the dosage should be strictly controlled in clinical use [137, 138]. Wu et al. [37] found that the total alkaloids of Aconitum tanguticum by gavage and intraperitoneal injection have certain acute toxicity to mice. It is preliminarily speculated that the target organs of poisoning are the autonomic nervous system and the motor nervous system, and the poisoning is fast and the duration is short. Although Tibetan medicine has good curative effect in the treatment of cholecystitis and cholelithiasis, its potential toxicity needs to be further studied. In our investigation and statistics, Compositae is the most commonly used drug for the treatment of cholecystitis and cholelithiasis, but the existing studies have found that some medicinal plants of Compositae have toxic effects. Lin et al. [139] forcibly fed the crude drug with the concentration of 0.24, 0.48, and 0.96 g/kg in Carthami Flos Decoction to pregnant rats, and found that the extract had toxicity to pregnant rats and their embryos, which could lead to abortion, weight loss, increase in kidney weight index, increase in embryo mortality, and intrauterine growth retardation (IUGR). Zhao Yunlong et al. [140] also confirmed the toxic effect of Carthami Flos on normal pregnant mice.

In conclusion, Tibetan medicine has research value in the treatment of cholecystitis and cholelithiasis, but its potential toxicity cannot be ignored. When using Tibetan medicine, we should pay attention to its dosage to prevent toxicity. In order to use the drug safely and effectively, the safety evaluation, pharmacokinetics, and toxicology of the drug should be studied.

## 5. Discussion

As a part of traditional Chinese medicine, Tibetan medicine is also an important part of the world's medicine treasure house. It has a long history, unique curative effect, and unique advantages in disease treatment. Tibetan people have accumulated a lot of medical treatment experience in the struggle between daily life and nature and diseases. At present, it is generally believed that Tibetan medicine has good curative effect on chronic diseases such as cholecystitis, cholelithiasis, hepatitis, high altitude polycythemia, gastritis, stroke, and rheumatism. In this paper, 170 kinds of Tibetan medicine and 38 kinds of Tibetan prescriptions were collected, and their families, genera, and medicinal parts were summarized. The results showed that these Tibetan medicines were mainly distributed in 24 families, among which Compositae was the most commonly used. In addition, herbs are the main source of these Tibetan medicines, and the whole herb is the most commonly used part.

At the same time, we should be aware of the gaps and limitations of Tibetan medicine research. Among 170 kinds of drugs for the treatment of cholecystitis and cholelithiasis, only 35 have been proved to have the related activity for the treatment of cholecystitis and cholelithiasis. Most of them have not been reported, and even a considerable number of drugs have not been studied in modern times. There are no literature studies on the use and safety of these drugs, so it is easy to cause problems when they are used. For example, Bolenggua is the second most frequently used Tibetan medicine in the treatment of cholecystitis and cholelithiasis. However, there is no report on the related effects of Bolenggua on cholecystitis and cholelithiasis. Similarly, Yanjing is used more frequently in prescriptions, and there is also a lack of related research. In addition, the drug components isolated from Tibetan medicine, such as anisodamine and matrine, have been proved to have the effect of treating cholecystitis and cholelithiasis, but the specific mechanism of action is not fully clear, and the possible synergistic effect between anisodamine and other components in the overall effect of Tibetan medicine has not been studied. At the same time, there are a considerable number of effective parts in Tibetan medicine, which are extracts and not monomers. The mechanism and components of action need to be further studied in order to achieve the controllability required by modern medicine.

To sum up, this paper provides information on the role of Tibetan medicine in the treatment of cholecystitis and cholelithiasis, and sorts out the drugs used by Tibetan medicine in the treatment of cholecystitis and cholelithiasis. In order to make better use of Tibetan medicine, we should, on the basis of traditional Tibetan medicine experience, use modern scientific means and methods to strengthen the research on pharmacology, phytochemistry, metabonomics, etc., evaluate its biological activity in vivo, identify its active components, clarify its mechanism of action, and clarify its toxicity and side effects, and/or perform clinical trial methods to conduct a new research on Tibetan medicine, and inject the connotation of the times into it, so that Tibetan medicine can become a standardized modern medicine [141].

## Figures and Tables

**Table 1:** Tibetan medicine for cholecystitis and cholelithiasis in modern research(the order of Tibetan medicine names is from high to low according to the frequency of use).

No.	Latin name	Chinese name	Tibetan name	Family	Medication part	Study on the treatment of cholecystitis and cholelithiasis and its complications	Reported biological activities associated with cholecystitis and cholelithiasis
1	*Swertia speciosa* D. Don	Yindu Zhangyacai	རྒྱ་ཏིག་	Gentianaceae	Whole grass	The crude extract was effective on the acute inflammation model. The high dose of benzene extract from gasoline extract could significantly reduce the edema of rat paws caused by carrageenan and formalin, and also reduce the area of turpentine-induced blisters compared with the control group [15, 16]. The purified mangiferin Vimang could significantly inhibit the edema induced by carrageenan and formaldehyde in rats, guinea pigs, and mice [17].	Vimang

2	*Swertia mileensis* T. N. Ho et W. L. Shih	Qing Yedan	ཏིག་ཏ།	Gentianaceae	Whole grass	Ethanol extract can promote bile secretion in rats, which has significant cholagogic effect [18]. The extract of total flavonoids has certain antibacterial effect on Glucococcus aureus, Bacillus subtilis, and Escherichia coli [19].	Total flavonoids

3	*Swertia mussotii* Franch.	Chuanxi Zhangyacai	ཟངས་ཏིག	Gentianaceae	Whole grass	Swertiamarin can significantly up regulate bile acid detoxification enzymes and transporters, increase the water solubility of hydrophobic bile acids, and eliminate bile acids [20, 21]. The compounds isolated from n-butanol can inhibit E. coli, Pseudomonas aeruginosa, Streptococcus faecalis, proteus, Salmonella typhimurium, and other pathogenic bacteria of acute cholecystitis [22–26].	Swertiamarin and kaempferone compounds

4	*Swertia punicea* Hemsl.	Zihong Zhangyacai	ཟངས་ཏིག	Gentianaceae	Whole grass	It has obvious inhibitory effect on xylene-induced ear swelling in mice, and also has certain inhibitory effect on the increase in vascular permeability in mice induced by acetic acid, and has a certain dose effect relationship [27, 28]. Kaempferone glycosides have good anti-inflammatory activities [29].	Kaempferone compounds
5	*Swertia cincta* Burkill	Xinan Zhangyacai	ཏིག་ཏ།	Gentianaceae	Whole grass	Water decoction can promote bile secretion, increase bile flow, with cholagogic effect [30].	Decoction

6	*Punica granatum* L.	Shi Liu	སེ་འབྲུ།	Punicaceae	Seed	Garnet acid has significant antiinflammatory effects, including increasing the level of 8-isoprostaglandin F2*α* in human body [31], the dependence mechanism of PPAR*γ* PPAR*δ* [32], inhibiting the activity of NF-*κ*B [33], inhibiting the production of ROS induced by TNF, and retaining the reaction induced by formyl methionine leucyl phenylalanine (fMLP), so as to achieve anti-inflammatory effect.	Garnet acid

7	*Aconitum tanguticum* (Maxim.) Stapf	Ganqing Wutou	བོང་དཀར།	Ranunculaceae	Whole grass	All kinds of extracts have good inhibitory effect on Gram-positive bacteria such as Staphylococcus aureus, drug-resistant Staphylococcus epidermidis, Enterococcus faecalis, Bacillus subtilis, and Bacillus cereus, Gram-negative bacteria such as Escherichia coli and Pseudomonas aeruginosa, and fungi such as Candida albicans [34]. Total alkaloids can inhibit anti-inflammation by inhibiting COX-2 [35], inhibit LPS-stimulated peritoneal macrophages to release NO and IL-1 [36], and inhibit xylene-induced ear swelling and subcutaneous agar granuloma in mice and acetic acid-induced writhing, and hot plate-induced pain in mice [37].	Alkaloid
8	*Hypecoum erectum* L.	Jiao Huixiang	པར་པ་ཏ།	Papaveraceae	Whole grass	The water decoction has inhibitory effect on Mycobacterium tuberculosis and Bacillus subtilis [38]. The extracts of cumin and chloroform can alleviate the pain caused by hot plate in mice, the ear swelling caused by xylene in mice, and the increase in peritoneal capillary permeability caused by acetic acid in mice, which can alleviate the inflammation and swelling in mice of different models [39, 40]. Ethanol extract can inhibit the formation of cotton ball granuloma and carrageenan-induced paw swelling in rats [38]. Ethanol extract can reduce LPS-induced inflammation in mice [41]. Proopioid (1) can inhibit LPS-induced inflammation of rat macrophages (raw 264.7), reduce LPS-induced NO production, and inhibit the levels of cyclooxygenase-2 (COX-2) and prostaglandin E2 (PGE2) [42]. Alkaloids can inhibit Staphylococcus aureus, Bacillus subtilis, and Escherichia coli [43].	Alkaloid

9	*Aconitum naviculare* (Brühl.) Stapf	Chuankui Wutou	བོང་དཀར།	Ranunculaceae	Whole grass	Total alkaloids can inhibit xylene-induced ear swelling in mice, acetic acid-induced increase in peritoneal capillary permeability in mice, and resist yeast polysaccharide A and carrageenan-induced paw swelling in rats in a dose-dependent manner [44].	Total alkaloids
10	*Sus scrofa* domestica Brisson	Zhu	ཕག་པ།	Suidae	Dung charcoal	It can effectively inhibit the irritation pain induced by glacial acetic acid and the ear swelling induced by xylene in mice by reducing histamine secretion and affecting arachidonic acid metabolism [45, 46]. Pig bile powder, pig bile acid, and its salts have varying degrees of antibacterial effect on a variety of bacteria [47]. Pig bile powder also has varying degrees of antibacterial effect on Streptococcus A and B, Staphylococcus aureus, tetracoccus, catarrhalis, dysentery, and Salmonella [48]. The ethanol extract can inhibit the growth of Escherichia coli and pneumococcus. Taurine can kill and inhibit viruses, mycoplasma, and bacteria [49]. Bile or bile salts can stimulate bile secretion after oral administration [50].	Taurine, bile salts

11	*Gentiana macrophylla* Pall.	Qin Jiao	ཀྱི་ལྕེ་ནག་པོ།	Gentianaceae	Flower or whole grass	KHU14 has inhibitory effect on ear edema caused by croton oil, foot edema caused by carrageenan, and capillary permeability caused by acetic acid [51].	KHU14

12	*Gentiana crassicaulis* Duthie ex Burkill	Cujing Qinjiao	ཀྱི་ལྕེ་ནག་པོ།	Gentianaceae	Flower	Polysaccharides have anti-inflammatory activity [52].	Polysaccharide

13	*Gentiana straminea* Maxim.	Mahua Qinjiao	ཀྱི་ལྕེ་དཀར་པོ།	Gentianaceae	Flower or whole grass	The volume of ear swelling induced by xylene in mice and the swelling rate of carrageenan toes in rats were reduced; the pain threshold of mice induced by the hot plate method was increased and the latency of mice induced by photoelectric tail flick method was prolonged; the number of writhing induced by acetic acid was significantly reduced [53, 54].	
14	*Rosa laevigata* Michx.	Jin Yingzi	རོང་སལ།	Rosaceae	Flower	Water extract and stem water extract can reduce the writhing times of acetic acid-induced pain in mice, improve the pain threshold of light heat tail pain in mice, inhibit xylene-induced ear swelling in mice and carrageenan-induced paw swelling in rats [55]. Ethanol extract significantly promoted the growth of granulation tissue and significantly reduced the total amount of leukocytes in pleural fluid induced by carrageenan [56].	Water extract, alcohol extract

15	*Nitrokalite*	Xiao Shi	ཟེ་ཚྭ།	Nitre family	Mineral	It can inhibit the formation of stone matrix, promote the dissolution of stones, and enhance the power of stone discharge, that is, inhibit, dissolve, and discharge stones [57].	Original mineral

16	*Ixeris chinensis* (Thunb.) Nakai	Zhonghua Kumaicai	རྩ་མཁྲིས་དམན་པ།	Composite family	Whole grass	Ethanol extract can effectively inhibit xylene-induced ear swelling in mice, carrageenan-induced paw swelling in rats, acetic acid-induced peritoneal capillary permeability in mice, and acetic acid-induced writhing in mice [58, 59].	Ethanol extract
17	*Selenarctos thibetanus* G. Cuvier	Hei Xiong	དོམ།	Family Ursidae	Gall, meat, bone, and fat	Bear bile powder has certain analgesic effect, can improve pain threshold, reduce the number of twists, and inhibit xylene-induced ear swelling [60]. It has obvious antibacterial effect on Staphylococcus aureus and Escherichia coli [61]. It has the effect of dissolving stone on gallstone, cholesterol stone, and mixed stone. It can reduce cholesterol content in bile, increase total bile acid content, and significantly improve the composition of adult bile. The results showed that the incidence of cholelithiasis was reduced, the content of free cholesterol in bile was reduced, the content of total bile acid was increased, and the effect of preventing the formation of bait gallstones was prevented. Goose deoxycholic acid and ursodeoxycholic acid can reduce cholesterol content in bile, which is mainly achieved by inhibiting cholesterol absorption in the small intestine and inhibiting activity of methylglutaryl CoA reductase, thus reducing cholesterol synthesis [62, 63].	Deoxycholic acid

18	*Ursus arctos* Linnaeus	Zong Xiong	དྲེད་མོང་།	Family Ursidae	Gall, palm, bone, and fat	The same as above	Deoxycholic acid

19	*Meconopsis quintuplinervia* Regel.	Wumai Lvronghao	ཡུངླལ་སྔོན་པོ།	Papaveraceae	Whole grass	Total alkaloids have obvious antiinflammatory effect on LPS-induced acute inflammation model mice, which may be related to the inhibition of proinflammatory factor TNF- *α*、 The expression of IL-6 is related to the production of NO and the decrease in the iNOS activity [64].	Total alkaloids
20	*Caragana tangutica* Maxim. ex Kom.	Qinggan Jinjier	མཇོ་མོ་ཤིང་དཀར་པོ།	Ursidae	Gallbladder	Ethyl acetate extract can inhibit xylene-induced ear edema in mice, carrageenan-induced foot swelling in rats, and acetic acid-induced writhing in mice [65].	Ethyl acetate extract

21	*Saxifraga tangutica* Engl.	Tanggute Huercao	སུམ་ཅུ་ཏིག	Saxifragaceae	Whole grass	The total flavonoid can significantly inhibit the ear swelling caused by xylene, significantly inhibit the enhancement of capillary permeability in mice induced by glacial acetic acid, and effectively inhibit granuloma in mice [66], which has the inhibitory effect on Staphylococcus aureus and Bacillus subtilis [67].	Total flavonoids

22	*Corydalis adunca* Maxim.	Huilv Huangjin	བ་མུང་ཟིལ་བ།	Papaveraceae	Root tuber	The water extract showed significant analgesic and anti-inflammatory activities in mice with acetic acid writhing test, hot plate test, mouse ear swelling induced by p-xylene and rat cotton ball granuloma model [68]. Total alkaloids can inhibit xylene-induced ear swelling in mice, agar-induced foot swelling in mice, inhibit sponge granuloma in rats, reduce the times of acetic acid writhing reaction in mice, and increase the pain threshold, which is an effective part of Corydalis pallidus for analgesia and antiinflammation [69].	Total alkaloids

23	*Gentiana striata* Maxim.	Tiaowen Longdan	སྤང་རྒྱན་དཀར་པོ།	Gentianaceae	Whole grass	Ethyl acetate extract has obvious inhibitory effect on xylene-induced ear swelling in mice [70].	Ethyl acetate extract
24	*Gentianopsis paludosa* (Hook. f.) Ma	Shisheng Bianlei	ལྕགས་ཏིག་ནག་པོ།	Gentianaceae	Whole grass	Water extract and alcohol extract have anti-inflammatory and analgesic activities [71]. Oleanolic acid and kaempferone have inhibitory effects on Pseudomonas aeruginosa, Bacillus megaterium, Staphylococcus aureus, Octococcus, Escherichia coli, Proteus, and Clostridium [72, 73]. The effective part of cholangiolysis by the bile duct drainage method is kaempferone [74].	Oleanolic acid and kaempferone

25	*Sonchus wightianus* DC.	Qu Maicai	མེ་ཏོག་སེར་ཆེན།	Composite family	Whole grass	Ethanol extract can effectively inhibit xylene-induced ear swelling in mice, carrageenan-induced paw swelling in rats, acetic acid-induced abdominal capillaries in mice, and acetic acid-induced writhing in mice [58, 59].	Ethanol extract

26	*Abrus precatorius* L.	Xiang Sizi	མདའ་རྒྱས།	Leguminosae	Seed	Leaf extract can significantly reduce the inflammatory reaction induced by croton oil [75]. Two triterpenoid saponins and their acetate derivatives in the extract have good antiinflammatory effect on croton oil-induced ear inflammation in rats [76]. Acacia alkaloid intraperitoneal injection significantly inhibited the local inflammatory reaction induced by Staphylococcus and inhibited the auricle swelling induced by croton oil in mice [77]. Clinical application found that it was effective in the treatment of jaundice after choledocholithiasis [78].	Triterpenoids, alkaloids, and anthraquinones
27	*Anisodus tanguticus* (Maxim.) Pascher	Shan Langdang	ཐང་ཕྲོམ་ནག་པོ།	Solanaceae	Root, seed, and rhizome	Anisodamine can promote the spontaneous discharge of choledocholithiasis [79, 80], promote bile excretion, and reduce the level of serum endotoxin. The mechanism of action may be related to the improvement of Oddi sphincter function after operation [81]. Anisodamine hydrobromide injection has the effect of promoting the discharge of a single CBD stone with a diameter of less than 10 mm [82].	Anisodamine

28	*Bupleurum chinense* DC.	Chai Hu	ཟི་ར་སེར་པོ།	Umbelliferae	Root	The extract can increase the bile flow of rats, promote the bile secretion in mice, and promote the secretion of total bilirubin in bile [83,84]. Ethanol extract and saikosaponin can promote the secretion of bile acid [85].	Saikosaponin

29	*Bupleurum marginatum* Wall. ex DC.	Zhuye Chaihu	ཟི་ར་དཀར་པོ།	Umbelliferae	Root, whole grass	Water extract and alcohol extract can increase the tolerance of mice to hot plate and writhing pain [86, 87] and can reduce the degree of ear swelling induced by xylene and the degree of toe swelling induced by egg white [88–90].	Saikosaponin

30	*Felis lynx* Linnaeus	She Li	གཕི།	Felidae	Gallbladder	Lynx bone has inhibitory effect on xylene-induced ear swelling in mice, carrageenan-induced foot swelling in rats, cotton ball granuloma inflammation model, and has relieving effect on hot plate pain and writhing pain [91].	Bone

31	*Solanum lyratum* Thunb. ex Murray	Bai Ying	wuluzuma	Solanaceae	Aboveground part	Saponins have antiinflammatory activity [92].	Saponins
32	*Solanum nigrum* L.	Long Kui	wuluzuma	Solanaceae	Aboveground part	Decoction has some inhibitory effects on Staphylococcus aureus, typhoid bacteria, proteus, E.coli, Pseudomonas aeruginosa, and cholera. Solanum can reduce the sensitivity of experimental animals to pain stimulation, and inhibit the development of rabbit ear scald or experimental foot swelling in rats [93]. Its salicylate and aconitate also have strong analgesic effects [94].	SolasodirIe

33	*Solanum americanum Mill.*	Shaohua Longkui	wuluzuma	Solanaceae	Aboveground part	It has anti-inflammatory activity [95].	Extractive

34	*Sophora davidii* (Franch.) Skeels	Bai Cihua	jiebeizhaibu	Leguminosae	Seed	It can inhibit the ear swelling and toe swelling of mice and reduce the permeability of capillary in mice [96, 97]. Matrine has an antagonism effect on the inflammation of earshell induced by croton oil in mice and rats, inflammation induced by carrageenan, and exudative inflammation induced by intraperitoneal injection of glacial acetic acid in mice [98].	Matrine

35	*Taraxacum mongolicum* Hand. -Mazz.	Pu Gongying	ཁུར་མང་།	Ccomposite family	Whole grass	The extract has significant anti-inflammatory effect and broad-spectrum antibacterial effect on a variety of pathogenic bacteria [99, 100]. Ethyl acetate extract can increase bile excretion [101].	Extractive
